# Supplementary figures and images for: Spittlebugs of Mediterranean Olive Groves: Host-Plant Exploitation throughout the Year
Source: Insects. 2020 Feb 18;11(2):130. doi: 10.3390/insects11020130 (PMC7074542; doi:10.3390/insects11020130)

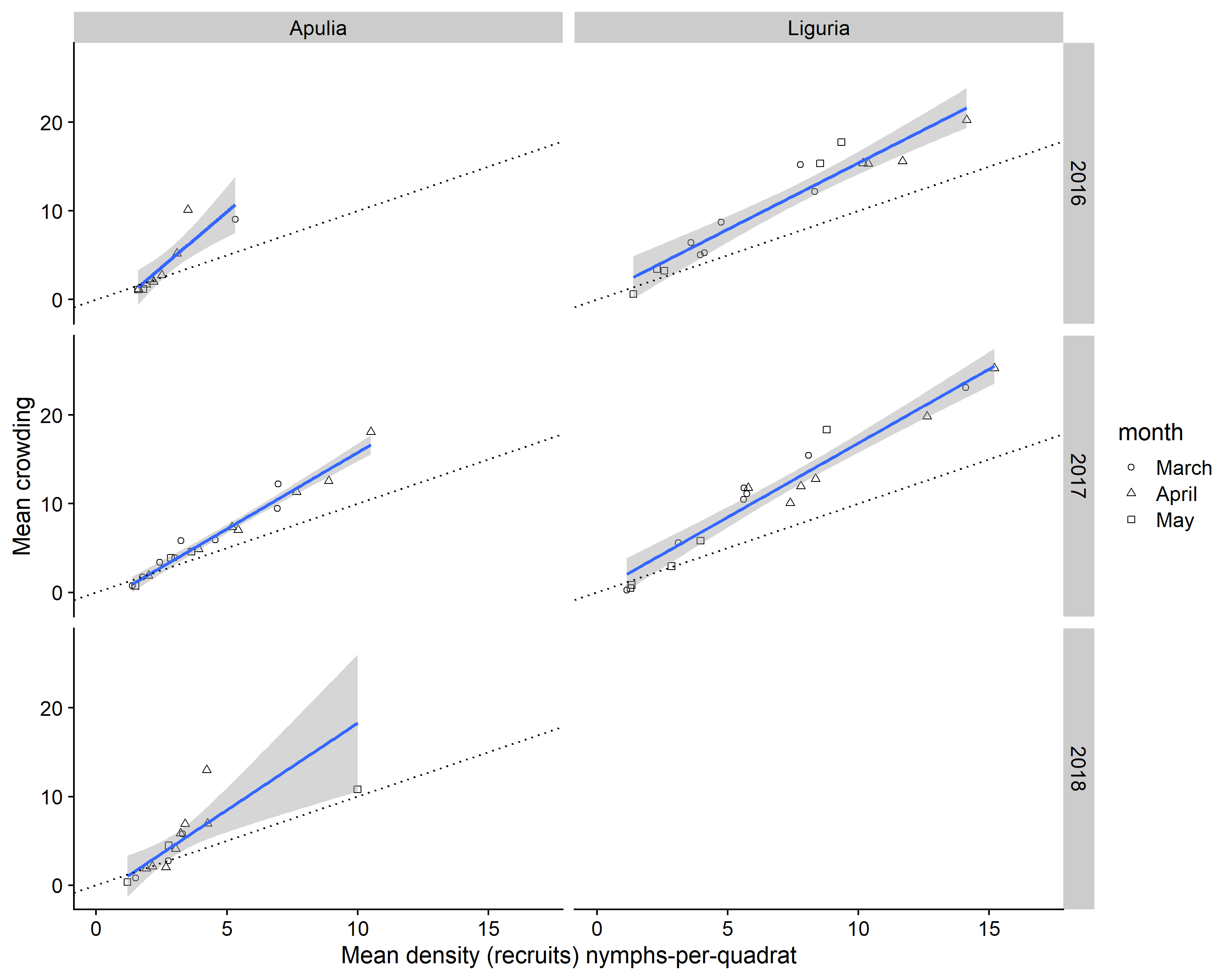

Supplement: Supplementary file 1 [file insects-11-00130-s001.zip › Figure S1.tiff]
